# The Cholera Epidemy at the Island of Fogo, in the Cape de Verds, in 1855

**Published:** 1857-10

**Authors:** José Fernandez Da Silva Leaō

**Affiliations:** Surgeon in the Portuguese Navy, and Colonial Surgeon of the Cape de Verd Archipelago; Corresponding Member of the Epidemiologica Society of London.


### TRANSACTIONS

OF THE

### EPIDEMIOLOGICAL SOCIETY OF LONDON.

### Papers and Communications.

### ON THE CHOLERA EPIDEMY AT THE ISLAND OF FOGO, IN THE CAPE DE VERDS, IN 1855.

By JOSÉ FERNANDEZ DA SILVA LEAÕ, Surgeon in the Portuguese Navy, and Colonial Surgeon of the Cape de Verd Archipelago; Corresponding Member of the Epidemiological Society of London.

[*Translated from the Portuguese by* GEO. MILLER, and J. O. McWILLIAM.]

THE island of Fogo lies between 14·42° and 15·1° North, and 24·8° and 24·32° West of Greenwich; consequently it is twelve leagues from north to south, and fourteen from east to west; and it is nearly circular. The island has several minor mountains, and in the centre a high ridge (*sierra*), traversing nearly its whole length, approaches the sea towards the north, assuming a semilunar form, the horns of which face eastward. In the centre of this figure there is a mountain of considerable altitude: it is, indeed, a volcanic cone, whose summit is the crater of the volcano, and it can be seen most conspicuously from the east end of the island. Some plains, which the natives call "*ahadas*", are seen here; but generally the declivity is uninterrupted from the base of the mountain ridge to the sea shore, there terminating in abrupt rocky fragments, here and there margined by a narrow strip of a dark coloured sand. The southern part of the island is more level, and the amount of flat is greater here than at any other part. The whole island is intersected by deep ravines, which, running from the summit of the mountain ridge to the sea, carry off the rains nearly as rapidly as they fall. Nowhere is there any morass to be found. The most abundant springs issue directly from the rocks, generally but little above the level of the sea; they supply excellent water, which being, however, carried away in imperfectly tanned goatskins, rubbed inside with the oil of the Pulga nut, has imparted to it a repugnant smell and disagreeable taste.

The lavas that have, at different periods, been emitted from the volcanoes, particularly those on the north side of the island, have destroyed cultivation; and, as there has been an utter disregard of an old law that no one should fell a tree unless he had previously planted two others, the north side shows scarcely a single tree, not even the wild fig or the tamarind. This want of vegetation probably contributes to the great scarcity of rain on the island of late years. During the periodic rains which prevail from July till November, the winds are from S.E. to S.W. In September and October, there are frequent thunder-storms; from December to June—the dry season—the wind ordinarily blows from N.E., and sometimes from N.W., more particularly in March and April, when the breezes are strong. Sometimes, during the rainy, as well as during the dry season, the wind blows from the eastward for two, three, four, and even five days together, and, by its dryness and rapid evaporating power, it is extremely disagreeable. It is the Harmattan from the African continent, the influence of which extends from 15° North latitude to 1° South. Remittent and intermittent fevers are reported to yield to its influence, and vaccine virus is said to lose its power during the prevalence of this wind.

The heat is excessive during the rainy season, particularly on the sea shore; but, during the dry season, the temperature falls, so as to cause a sensation of cold during the months of December and January.

The population of Fogo in June 1855 was 13,101. It is divided into parishes, of Nostra Senhora da Conceiçao, S. Lorenzo, S. Katharina, and Nostra Senhora da Ajuda, commonly called Monteiros.

The parish of Nostra Senhora da Conceiçao is bounded on the south-east and west by the ocean, and from the north to the west by the parish of San Lorenzo, and to the east by Mount Disimo. The town of San Philip is the largest town on the island, and the only place where there can be said to be any commerce. Here is the best anchorage, and here the principal families, nearly all of whom are of European origin, reside. The population of San Philip was 258 free males and 297 free females, 332 male and 337 female slaves; these, with 1,257 free men, 1,430 free women, and 49 male slaves, and 43 female slaves, distributed over various parts of the parish, give a total population for Nostra Senhora da Conceiçao of 4,043.

The district of San Lorenzo, the most extensive and populous of the island, is situated about the centre of it, a little westward. It is bounded on the east by the mountain ridge; on the west, by the sea; on the north, by the Ozoria ravine, which separates it from the district of Nostra Senhora da Ajuda. The population consists of 5,195 persons; namely, 2,279 free men, 2,426 free women, 236 male and 255 female slaves. This population is exceedingly scattered

To the east of this great ridge, and on the opposite side of the preceding district, is that of San Katharina, bounded on the north by Nostra Senhora da Ajuda and the ravine of Balea. This district, although nearly as large as that of San Lorenzo, has only 712 inhabitants; viz., 354 free men and 355 free women, 1 male and 2 female slaves. The greater portion of this district is devoted to pasturage. The inhabitants are spread over the whole district. At a place called Casinha, which is on the sea shore to the north of Alcatraz, there are a few ruined houses surrounding a church.

Finally, at the north of the island lies the district Nostra Senhora da Ajuda, with a population of 3,151; namely, 1,446 free men, 1619 free women, and 30 male and 56 female slaves. This population is formed into larger groups than the preceding districts, consisting of small villages near the sea shore; their names are, Sumbango, Feijāosinho, Sitio da Igreja, and Atraz.

The soil of Fogo is capable of growing not only such produce as is usual to its latitude, but many that are European-Indian corn, beans, cassava, and of late years the sweet and the common potato; ground nuts and sugar-cane have of late years been chiefly cultivated. Of the large vineyards and cotton-fields that formerly existed, there is now scarcely a vestige. There are few goats or swine, but there is a large quantity of horned cattle; indeed, in years of abundance, the pasturage is barely sufficient for their support; and, in years of drought, many of them perish of hunger, while others, impelled by want of food, leave their herds, and devour the crops on the cultivated lands: so that, in fact, in those seasons of scarcity, it is not the want of rain alone that does mischief, but the destruction of the crops by the famishing cattle; and this will continue to be the case until the number of cattle in the island is in proportion to its pasturage.

All the natives of the island are cultivators of the soil; but, with very few exceptions, they know nothing of agriculture, and are so indolent that they content themselves with merely scratching the earth. They work for two or three months in sowing and reaping; the rest of the year is passed in eating, smoking, and sleeping. An industrious man is a "rare bird." They live chiefly on Indian corn, beans, cassava, potatoes, fish, milk, and occasionally goat's flesh. Bread and beef are enjoyed by the upper classes only; and the only houses with an upper story, or any pretensions to comfort, are at San Philip. In the other parts of the island, you find low, damp, ill built, unwhitewashed huts, some of stone, covered with grass, and floored with slabs; and in such as these you will find living men who call themselves "*morgados*" (lords of the manor), and, although living in pigstyes, make pretensions to be thought rich.

The island of Fogo is one of the most salubrious of the Cape de Verd Archipelago, and to its climate many owe the restoration to health of their system, shattered by the country fevers (febres palustres); phthisical patients, the temporary suspension of the march of the disorder. However, notwithstanding so much salubrity, the island is subject, like all others of the Archipelago, to an endemic of a bilious nature, which the natives call "*lavadias*", or the "May sickness", which manifests itself by red urine, tongue furred, yellow, bitter taste, vomiting and dejections bilious, and which ordinarily, after a course of three or four days, terminates in recovery, without the aid of medical treatment. It rarely happens that a case assumes a dangerous form, and much more rarely that death ensues.

But, besides this annual endemic, in the months of October and November, from the sea shore in the parish of San Lorenzo to the heights of San Jose, cases of remittent and intermittent fevers manifest themselves, and their amount and intensity are generally in proportion to the abundance or scarcity of the rains. However, let this locality be affected as it may in this way, the neighbouring district of Nostra Senhora da Conceiçao is very rarely so, except at a place called Correia, where occasionally fever of one or the other form shows itself.

In the year 1854, the rains were scanty in all parts of the island, especially in the Nostra Senhora da Conceiçao district.

The sanitary state of the island was as usual, and there were but few cases of country fever in the months of October and November. The island maintained the same state of salubrity in 1855, until the month of July, having almost imperceptibly passed through the May endemic, and without the appearance of any epizootic or epiphytic disease.

Such was the sanitary state of Fogo when, on the 29th of June, there was seen, to the southward of the island, a barque, that during the following night, with a light hoisted at her masthead, kept tacking between Fogo and Brava. In the morning she was to windward of the port, with her flag flying. There was at the same time in sight the sloop *Benjamin*, which, on the previous day, had sailed from the principal port—the port of the town—for the Salt Port. The barque, now at the northern side of the Rombo Island, got within hail of the sloop, and asked at which of the ports in the islands, that were in sight, provisions and water could be most easily procured. When it was answered that Villa da Praia, in San Jago, was the best, the captain not having charts of the islands, the bearings of that port were given him by the sloop. Following the directions thus obtained, the captain continued to beat to windward, so as to weather the north point of Fogo; but, on one of the tacks, he so neared the land, at the valley of the Cavalliero, that the harbour master of Fogo, who was out in his boat, and had already endeavoured to communicate with the barque, rowed alongside, and, jumping on board, assured the captain that all the necessary supplies were procurable at Fogo. He then piloted the barque into the anchorage.

The barque having dropped anchor at 2 P.M. of June 30th, 1855, the harbour master sent on shore to the deputy of the Board of Health the ship's bill of health, by which it appeared that the Sardinian barque *Corça*, of 273 tons, was commanded by Peter Bozano, with a crew of twenty-two men; that she sailed from Savona on the 31st of May, on a voyage to Monte Video and Lima; and, from an unsealed document in manuscript attached to the bill of health, it further appeared that she had 240 passengers on board, but there was no mention of these in the bill of health. The officer in charge of the sanitary office at the port deemed that the vessel having what is called a clean bill of health was sufficient to justify his admitting her to *pratique*, and he did not visit her. The barque was in due course visited by the customs authorities; and a tidewaiter, called Anselmo Pires, was left on board of her. The captain came at once on shore, and consigned his ship to John Barboza, merchant; and requested leave from the Board of Health and the collector of customs to land his passengers and their baggage on the following day, intimating at the same time that he had put into the island for fresh provisions and water.

On the 1st of July, one hundred and nineteen passengers were landed from the barque, and lodged in three central houses in the town of St. Philip, and some in the barracks. On this day, and the day following, the contents of many mattresses were thrown overboard from the ship—so many that the inhabitants of the island could see a large space of sea covered with the floating straw, which, in process of time, was washed upon the beach. Besides these, the passengers, as they were landing, emptied many more mattresses; yet each passenger still possessed a mattress. It may be asked how all this came to pass? To whom could the emptied mattresses belong? The captain of the ship, moreover, seeing some of the natives of the island collecting the straw, told them not to make use of it, as it was full of vermin; but on their replying that he was mistaken for it was quite clean, he rejoined that they might do as they pleased, but that they would do well not to use it.

Among the passengers first landed there was a woman with an infant at her breast, that swooned away, and was, to all appearance, dead; and the mother at once returned to the ship with her apparently dying child. Such, indeed, was the sickly look, the lowered condition, and the weakened form of many of the passengers, that they walked about assisted by their fellows. Frequently, men and women were observed to stop and answer the call of nature in the public streets, having no time to seek seclusion for this purpose; and such was the intensity of their thirst, that they went from door to door in the town begging for water. These circumstances and some others attracted the attention of the inhabitants, and caused them to fear visits from such guests.

Many of the inhabitants were, however, so moved with compassion at the sight of so many persons roaming about the streets in distress, that they gave them shelter. Amongst those benevolent minded persons, the daughters of Mr. Alexander Jose d'Abreu were conspicuous; and seeing a poor woman pass their door with two children, so moved were they, that they called them in; and while one of these ladies was in the act of taking the youngest child in her arms, it was attacked with violent vomiting and purging, and fell into a state of utter prostration. The mother at once fled; and, returning with her husband, both expressed, by gesture, what intense anxiety the ailment of their child caused them. The only surgeon in the place was immediately called to the child, which had vomiting and diarrhœa, and was for some days in a dangerous state, but eventually recovered. It was in Mr. Abreu's house that the child was attended to. His eldest daughter and her slave woman, "Noberta," nursed it, and the latter washed the linen soiled by the child.

All the members of Abreu's family remarked that the character of the evacuations, and of the vomited matter, seemed most to attract the attention of the child's parents. Two other children were also seriously attacked immediately on landing—one of them on the beach, and the other a short time afterwards. Whence could this illness have arisen? It could not certainly be attributed to anything in the island, because one of them was attacked before landing, and the other in a very short time afterwards; and also because every one, old and young, on the island was in the enjoyment of good health.

On the 2nd of July the remainder of the passengers landed, and their appearance denoted that they belonged to a better class of society, and they were also in better health than the others; they shunned communication with those who were landed on the first day. Some of them lodged on shore, while others came on shore during the day, but returned to the ship at night.

The captain authorised his consignee to diet one hundred and sixty-six passengers; and although he allowed beef, mutton, rice, and wine, he rigorously objected to their being served with fish, green vegetables, or fruit, and begged that these should not be shown them, in case they should be tempted to eat them. Now, all who know anything of a long sea voyage, are aware with what avidity vegetables and fruits are sought for and devoured. Yet these people, after thirty days passage, refused them.

The nauseating odour issuing from the houses occupied by the passengers, having been complained of by the neighbouring inhabitants, a visit was paid them by the deputy of the Board of Health; and the result of that visit is thus described by the naval surgeon of the first class, Guilhame Maria Mayer: "It coming to the knowledge of the now deceased delegate of the Board of Health that some of the passengers suffered diarrhœa, and that there was great want of cleanliness in the houses they occupied, he proceeded to inspect them, accompanied by the Clerk of the Council; and not only did he find want of cleanliness, but also an adult and child affected with diarrhœa; after giving instructions for cleansing, he retired. On the next day another inspection was made, but neither the man nor the child with diarrhœa was there; both of them had been removed to the ship."

On the 4th of July four of the passengers attended at mass; and in the middle of this religious rite one was suddenly attacked with vomiting. The surgeon was immediately sent for. He saw the man, and prescribed for him, when he was conveyed to the ship, but did not return to the shore. On this same day another passenger begged water from Mr. Thedem Monteiro for his son, who had just been attacked with the sickness. Mr. Thedem afterwards asked for this child of its parents; but the reply was, he had been sent on board. In this way children disappeared from day to day.

It is necessary now to inquire what was going on on board the ship while these things were passing on shore. Anzelmo Pires, the customs tide-waiter, who was on board during the whole period of her stay at Fogo, informed me that there were two classes of passengers on board; of the first class—about twenty in number—all lived with the captain in the after part of the ship. The other class, much larger in number, were located in the hold, which was fitted up for passengers. Some among the first class looked sickly; but among the second class many looked very ill, and were suffering much from diarrhœa, for they kept going to the privies on board continually. The greater part of the second class passengers landed on the 1st of the month, and the remainder with those of the first class on the second, except ten first class and fifteen second class, who remained on board. The tide-waiter also said that the crew of the vessel had a separate compartment, and that none of them appeared to have suffered, except the mate; that those who came on board from the shore ill immediately went to the hold; that he frequently saw passengers lying on the decks in agony at night, and that in the morning there was nothing to be seen but their mattresses rolled up. He further corroborated the evidence of the people on shore, with regard to the mattresses being emptied into the sea after the passengers were first landed. He added that the captain caused the hold to be thoroughly whitewashed; and that on the 5th, when the vessel was clean, some of the passengers came on board; and that on the afternoon of the 6th all the passengers precipitately returned on board, and the same night the *Corça* sailed.

From all the circumstances as narrated, it may be concluded, that when the vessel arrived at Fogo, there existed among the passengers an epidemic of a grave character, in which the predominant symptoms were vomiting and diarrhœa, of which some died on board : that the vessel, on her arrival at Fogo, had not the number of passengers (240) which she originally embarked, inasmuch as she landed 166 only, and, adding the 25 that remained on board, 49 remain to be accounted for : that every care was taken to conceal the nature of the disease under which the passengers laboured—the surgeon in the town having been but rarely consulted, although it was notorious that many were ill. The captain, moreover, immediately on anchoring, landed the passengers, and maintained them at his own cost, and at once proceeded to disinfect his ship : the first things he inquired for on shore were lime and vinegar in large quantities.

Based on information derived from the most impartial and competent persons of the town, I have narrated the state of the island before the arrival of the *Corça ;* I have told what was the health of the crew of that vessel; and I have detailed the nature of the intercourse between the ship and the shore. Upon the same authority, let me recount what occurred at this time, and afterwards, among the inhabitants of the Port San Philip.

On the 3rd of July, Noberta, the slave of Mr. Abreu, who, as we have seen before, had been nurse and washerwoman to a child from the ship who sickened in Mr. Abreu's house on the first of that month — fell herself sick. Noberta's symptom's were,—vomiting ; white coloured evacuations at first, afterwards watery or rice-watery ; evacuations at first fetid, and afterwards almost inodorous ; insatiable thirst ; suppression of urine ; dull appearance of the skin, particularly about the eyes, nose, and mouth ; rapid wasting of the body ; complete alteration of the features ; coldness of the skin ; profuse cold perspiration ; wrinkling of the skin of the hands and feet, as if they had been for some time soaked in water ; cramps in the legs, with pains and screaming ; excessive depression, but retention of the intellectual faculties ; change of the voice to a sound peculiar to the disease ; and death. On the following day, Claudio, the slave of Mr. Antonio Medina, who on the preceding evening had visited Noberta, and who lived on terms of intimacy with some of the passengers, and who, moreover, I am assured, had thrown the corpse of one of the passengers into the sea, assisted by Canuto, the slave of Mr. Vasconcellos—was attacked and died on the 5th, making the second case on the island. Canuto died in two or three days afterwards, after only a few hours suffering. On the 5th and following day, many persons of the lower orders, who had been in more immediate contact with the passengers of the *Corça,* were gravely attacked with the same disorder. On the 6th, it became known throughout the town that a number of people had been seriously attacked, for all seemed to be begging assistance for relations, friends, or slaves that had been taken ill. The whole town seemed to be given up to terror and confusion. What disease could this be? asked many, who, beginning to doubt the correctness of the designation given to it by the surgeon —who had reported that the May sickness, not having shown itself at its proper season, had *now* appeared, but in a graver form. After finding for it various extravagant names, some one who had seen the cholera elsewhere suggested to Mr. De Sousa, the surgeon, that it was that disorder (cholera morbus); but he immediately asked, what similarity was there between this disease and the cholera morbus? In justice to the attainments of my deceased colleague, I must say that I feel convinced that some humane motive led him to disallow the correctness of this diagnosis. As the day advanced, cases of death occurred one after the other. Some, who at daybreak discussed the nature of the incipient epidemy, and gave a helping hand to the sick, were themselves in a few hours attacked; some of them so gravely, that they succumbed to the disease the same day.

Terror struck and uncertain what to do, the inhabitants wandered about, seeing relatives and friends or slaves falling victims to the disease, and expecting that the same lot might be their own; when it occurred to Mr. Vasconcellos to embark in the brigantine *Cordialidade,* and abandon the island. This vessel was consigned to Mr. Vasconcellos, and had arrived on the 4th of July. This determination was very generally acceptable, and the rule became, "Save himself who can". The flight of the principal families was so precipitate, that parents abandoned their offspring, husbands their wives, and wives their husbands. Many indeed reached the *Cordialidade* with scarcely any clothing, and with no thought for provisions.

In contrast to the terror and confusion among the natives, the passengers of the *Corça* continued to walk about the streets of the town, when one of them inquiring at the house of Mr. Monteiro where he could purchase some eggs, he was advised that the best thing he and his companions could do was to be off, if they did not want to pay dearly for introducing into the island a disease that had already numbered so many victims. Within five minutes the whole of the passengers were in movement, and in two hours they had all re-embarked. The captain went on board without taking on board any fresh provisions or water, for which he at first said he had expressly come in. It was not the want of these things, but the critical sanitary state of his vessel, that caused him to put into the anchorage.

On the 6th of July, the day on which the *Corça* left, there were upwards of thirty new cases, which, being added to those of the preceding day and night, made ninety, of which by eight o'clock in the evening seventeen were fatal, and some of them quite suddenly so. Each moment the epidemy seemed to acquire greater intensity, attacking especially those slaves and tavern keepers who had been in more immediate communication with the people of the *Corça*.

Fatal cases occurring without interruption one after the other, some in a few hours, the cries of relatives and friends, the passing through the streets of the corpses of those who in the morning had risen full of life and vigour, added to the fact of this calamity having taken place in the most healthy part of the island, where nearly all are either related, or, at all events, personally known to each other, led many of those who had protested that they would not abandon the island, to seek refuge that same night on board the brigantine.

The captain of the *Cordialidade*, in accordance with the desire of the multitude that had flocked on board his vessel, got under weigh and proceeded to Brava, at daylight on the morning of the 7th of July, and arrived there at eight o'clock; but the authorities of that island, apprehending from the great number of passengers on board the vessel that some particular disorder had broken out at Fogo, refused her pratique, and the captain, unwilling to undergo quarantine, returned to Fogo, where he anchored at four in the afternoon. Fortunately the sloop *Benjamin* was still at anchor in the Salina when the *Cordialidade* left, and had in the meantime been ordered round to the harbour of San Philip.

The captain of the *Cordialidade* endeavoured in vain to get rid of his passengers, with the exception of four persons who went on shore, and a family that went on board the *Benjamin*. The rest remained on board the *Cordialidade*, in which, on the evening of the same day (7th), they sailed for Villa da Praia, in San Jago, where they arrived on the

9th. The sloop *Benjamin*, with a similar freight of the natives of Fogo, arrived at Villa da Praia on the following day. There were on board these two vessels collectively 38 free men, 65 free women, 20 male and 19 female slaves.

The governor-general having caused a temporary lazaretto to be erected on shore, the people from both vessels performed quarantine there, several cases of cholera occurring while under this detention.

While one portion of the inhabitants of San Philip were crossing the sea to avoid the epidemy, the others, who had not been stricken by the disease, fled to their miserable and confined huts in the interior of the island. All but the indigent, the moribund, and the sick, and some slaves, who, abandoned by their masters, were without food or succour of any kind, had fled. Many of these unhappy slaves were found lying and groaning in their masters' yards, with the pavement for their bed and the dome of heaven for their covering. Others were found that had evidently been dead for days, half-devoured by the famishing dogs and swine. What a horrible death must have been that of those unfortunate beings, who had endured not only the horrors of the disease, but with it the fulness of desolation! Unhappily others, who were not slaves, shared the same fate.

The interments were made by the slaves, but as most of them were attacked by the disease, and some of them suddenly while in the act of carrying corpses, they at length became so terrified that their masters were obliged to use their authority to make them perform their duty, giving them from time to time large quantities of rum. At length things came to such a crisis that corpses remained for days piled up at the entrance of the cemetery, at the church door, and in the houses, until carted away or dragged through the streets to their graves. The interments were at first in the cemetery, which is situated at a little distance from the town; but soon the number of dead so increased, that the bodies were consigned in various other parts of the town, and in the yards behind the houses.

While all this was passing in the town, the people in the country rendered no assistance; whole families were attacked, without a soul to help them, not even to fetch a drop of water from the nearest spring, which is two leagues distant. What could be more horrible than the want of water in a disease where the thirst is so insatiable? In such circumstances, what steps, it may be asked, did the authorities take? The Administrador do Concelho, and the military commandant, fled; the one on board the *Cordialidade,* and the other on board the *Benjamin,* abandoning the people that had been committed to their charge. It is difficult to ununderstand how those authorities could act so disgracefully, and throw away the opportunity of proving how such offices as theirs could be worthily filled. All honour to the parish priest, who, with the cross of our Saviour folded on his breast, went on his mission of charity by day and by night, administering the holy sacrament to the sick, and, with religious fervour, giving encouragement and consolation from door to door. Nor was the health officer, De Sousa, less worthy of eulogium, who alone, in the midst of this epidemy, with but few resources, consulted not his own safety, but that of others, passing days and nights at the bedside of the sick, doing all that man could do to rescue them from death. It was this noble surgeon who, exhausted, and indeed already morally killed by the epidemy, seeing his patients in torment from thirst, went forth from the town on the 10th to the Morgado Francisco, J. D. S. Monteiro, to beg that he would send water into the town. The benevolent, but aged and bedridden islander, had sufficient force of character to order his slaves to carry water into the town, and distribute it gratuitously among the sick. The surgeon, having succeeded in obtaining a supply of water for the sick in the town, was now called to attend some of those who had fled to the parish of San Lorenzo, and who had sickened there. Having seen many patients, this brave man was returning late at night to Pico Pires, with the intention of being in San Philip early in the following morning; unhappily, his last hour was nigh, for, in the course of a few hours, he himself was attacked, and fell on the 17th of July, like a valiant soldier, at his post. The only official person now left in the town was Mr. Justino Antonio da Silva, Secretary to the Municipal Council, who was indefatigable in his attention to the sick, and in burying the dead. Would to God that all the authorities had comported themselves as did this Justino! The authorities of San Lorenzo could not then have excused themselves from assisting San Philip, on the plea that their own authorities had fled to Villa da Praia.

During the time that the disease was at its height in the town of San Philip, the other parts of the island were in a most healthy condition. I inquired very minutely whether there had been any change in the habits, the food, or the moral condition, of the inhabitants; or any meteorological peculiarity; or any excavations or opening of graves, exposure of putrefying matters; or, in fact, any changes, personal or material, that could give rise to such an epidemy as that which affected the town of San Philip; but I could detect nothing, excepting the communication with the *Corça,* to account for its appearance there.

In the other parts of the district of Nostra Senhora da Conceiçao, as well as in the district of San Lorenzo, up to the time of the arrival of the fugitives from San Philip, the general health was good; but, in a few days, some of these fugitives began to sicken, and the epidemy then spread among the people of San Lorenzo, attacking those living with or nearest to the people who had come from San Philip. This fact can be vouched for by many now on the island; and it is equally a fact that several families evaded the disease altogether by flying to uninhabited districts, and shutting themselves from all communications from without. Senhor Jeronymo Jose do Sacramento Monteiro, with a portion of his family, and others, escaped in this way. At the same time, it must be admitted there were several persons who experienced no evil result, although they were in immediate contact with many who were severely attacked.

Such was the number of persons from San Philip who fell sick in the other parts of Conceiçao, and in that of San Lorenzo, that, in seven or eight days after the disease had appeared in San Philip, it was spreading over the whole district of Conceiçao, and part of that of Lorenzo. Now, let us see how the disease extended over Concciçao and San Lorenzo, beginning, in the first place, however, with the district of San Katharina.

Four of the slaves that had been abandoned in the town of San Philip, two belonging to Senhor Joacquim Monteiro de Macedo, both called "Jeronymo"; one called "Beafe", belonging to Senhor Gomes Barboza; and the fourth called "Surna", belonging to Senhor Benjamin Barboza,—became terrified at seeing their companions in the work of interment falling constant victims to the disease; and on the 8th they took a boat, with the intention, they said, of reaching their own country—Bissao. These unfortunate creatures, coasting along the south side of the island, arrived on the 9th close to the point of Alcatraz; but Jeronymo the elder was already suffering from constant vomiting and purging, and his companions, having carried him on shore, sheltered him under a tent made from the boat's sails, and repaired to a place in the neighbourhood, called Casinha, to obtain assistance for their comrade; but, before the people could reach him, he was dead. Jeronymo the younger, a few hours after arriving at Casinha, was attacked with vomiting, purging, cramps, etc., so severely, that he died on the following day. In three or four days, Manuel de Andrade, an inhabitant of Casinha, who had assisted to nurse the younger Jeromymo, was attacked with the same disease. This man had not been to the town for many days. On the 8th, the slave Manuel dos Reis, belonging to Senhor Marcellino Felix Medina, left San Philip for the San Katharina district. On the 9th, he was found by Sebastian Roderigues at Bomdardiera, lying on the ground, and so ill that Sebastian took him on his back to his house. The slave died, and Sebastian and his family were nearly all attacked by the same disease.

In Cova Matinha, the first person attacked by the disease was Henrique dos Ramos. He was a fugitive from San Philip, and had arrived the day previously. In short, all the information obtained led to the conclusion that the disease was communicated by the fugitives from San Philip to San Katharina, inasmuch as the fugitives were the first sick in San Katharina, and next in succession those who waited upon them, passing from family to family, until it got dominion over the whole district. At the same time, the slaves Surna and Beafe, having lost their two companions from cholera, directed their steps to the district of Nostra Senhora da Ajuda, where they remained during the whole of the epidemy without suffering themselves, although persons who had intercourse with them were attacked by the disease.

Finally, let us see who were the first to be attacked in the district of Nostra Senhora da Ajuda. Mr. José Vasconcellos' slave, Julio, left the town on the 7th of July; on the 8th, he reached Mount Queimado; on the 9th, he was attacked with vomiting, purging, cramps, etc.; and died on the 10th. Three days afterwards, the slave woman Maria, who nursed this Julio, and who for more than a month had not been near the town, sickened and died of cholera.

At Larango, a place near to Ingreja, Sumbango, and Feijãosinho, cases manifested themselves among the fugitive slaves from the town, and from them propagated to the respective inhabitants of those places.

At Ingreja, the first attacked was one Antonio, who had on several occasions been with the sick at Laranjo. He was attacked on the 12th, and died within six hours. Eight days afterwards, Feijãosinho, Sumbango, Laranjo, Ingreja, and Atraz, had many sick, and the disease was soon propagated over the whole island.

Who, on the 30th of June, could have foretold to the people of Fogo that their island, one of the most salubrious of the whole group, would, in a few days, be laid waste by a desolating epidemy; that, by the 12th of the following month, it would be so completely under the deadly dominion of this epidemy that many of its inhabitants, abandoning their nearest and dearest ties, would be seeking safety in San Jago?

On the 15th of July, Senhor Guilhame Maria Meyer, first class surgeon of the navy, arrived in commission on the island; and, in his report to me, I find the following:—

"As soon as I landed, I set about inquiring into the sanitary condition, not only of the town, but of the island generally; and I learnt that nearly all those who remained in the town were affected with purging and vomiting; that, on the previous days, there had been great mortality, amounting to seventeen and eighteen deaths daily; that, on the day preceding my arrival, there had been eight deaths; and, since my arrival, in the morning there had been five deaths; and lastly, that some of those sick were in great danger. The number of sick I visited that same night exceeded 100. In some houses I found as many as five or six sick, nearly all of them extremely poor. To describe the state of misery and abandonment they were in would be difficult; and I shall confine myself to saying that, in addition to cholera, they had to contend with hunger and thirst. Of the sick, some were just attacked, others had been ill a day or two, and a small proportion were convalescent. They had had no medical assistance for two days. My colleague De Sousa was lying dangerously ill at Pico Pires; and, indeed, he died in forty-eight hours after my arrival.

"Having finished my round of visits, I returned to my quarters, where I was occupied until 4 in the morning preparing medicines.

"I next obtained a house for a provisional hospital for all who applied, and by 8 in the morning all was ready. At 9 I began my rounds of visits, and the military commandant gave orders for the removal of patients to the hospital. On the occasion of the second visit, I found the patients in less depressed spirits. Occupation fast accumulated upon me; for, in addition to my patients in the town, I had constant applications from the country for advice and medicines. On the following day I inspected the localities where the dead had been buried, and found that the interments had been so ill conducted that in many places, especially where the graves had been opened of the churches La Misericordia and the parish church, the bodies were in fact serving as food for the dogs and swine. I caused the clothing of those who had died of the disease to be destroyed, and the houses well aired and fumigated."

In the foregoing report it will be seen what was the state in which the surgeon found the island, and the steps he took prior to my arrival on the 20th of July, when I arrived there in execution of a government decree.

Notwithstanding that what I had heard already from the Fogo fugitives at San Jago led me to believe that the disease that had driven them from Fogo was cholera, and that it had been imported, yet, when I landed at Fogo, I resolved not to be influenced by the opinion to which such information led; and I determined to inquire afresh, and judge for myself, and to arrive at a final conclusion from what I myself saw and heard from disinterested persons, on whom I could place reliance. It was upon this principle of action that I commenced the fulfilment of my mission.

Immediately after my arrival on the island, I visited all the sick in the district of Nostra Senhora da Conceiçao, and on the following day those of the other parishes. In all of them I found a greater or lesser number of sufferers from a disease that showed a perfect similarity of progress. One of the characteristics of an epidemy, says Schmurres, is, that just as a leaf of a tree shows the nature of the tree itself, so an individual case may show exactly the general character of an epidemy. I came to the conclusion that an epidemy raged throughout the island, which, from the march it had taken, and from what I observed, convinced me that, in the district of San Conceiçao, it was in its third stage of progress; in that of San Lorenzo, in its second; and in that of Nostra Senhora da Ajuda, in its first.

Not willing to form a precipitate diagnosis of the epidemy I was observing, notwithstanding that the individual cases presented symptoms so characteristically pathognomonic that it was sufficient to see a couple to determine with certainty what the disease was, especially as I had former experience of cholera, I still attentively watched the cases, and divided them, according to their gravity, in two classes. In the first and second I recognised all the symptoms and the march which the best authors have assigned to cholerine and to Asiatic cholera, from its invasion to the cyanic period, and to that of incomplete reaction; and being the same set of symptoms which came under my notice during the epidemy that devastated Portugal in 1833. From these facts, I am certain that the disease that reigned at Fogo in 1855 was the Asiatic cholera; and my medical colleagues in the commission are of the same opinion.

Having faithfully narrated the outbreak and spread of cholera over the whole island, I think I have given authentic proof that it was imported into the island by the Sardinian barque *Corça*.

While I was investigating the nature and origin of this epidemy, I at the same time prescribed for the sick, and put in practice various sanitary measures. Practice and theory had demonstrated to me, that the medical man who undertakes to combat an epidemy, has not only to look after those actually sick, but has also to attend to matters connected with public and private hygiene, the omission of such attention frequently rendering the disease more fatal and of more prolonged duration, and, indeed, occasionally giving rise to other diseases. I therefore began by inspecting the residences of those public *employés* who had preceded me in the inquiry,—the Provisional Hospital and the Barracks; and, finding these *employés* all huddled up in one room, that served the double purpose of a drug room and a residence, I ordered that they and the troops, who were improperly quartered, should be suitably accommodated, and within the reach of immediate medical aid. In the hospital I arranged that more space should be given to the sick, and the drug room placed in the same building.

I at the same time issued sanitary regulations, and saw that they were attended to. I caused the graves to be dug to a greater depth, as many of those already used were not sufficiently deep, so that the putrifying corpses emitted the most vile effluvia. I caused the houses to be disinfected and ventilated.

Personally superintending this work, I frequently found in the empty houses vessels containing excrement and vomited fluids of those who had died, and their clothes and bedding just in the state in which they had been left.

After putting these hygienic matters in train, I handed over the charge of the district to Mr. Mayer, and established myself in the centre of San Lorenzo, where the epidemy was then at its height; and I did all in my power to encourage and put spirits into those who were not attacked, to minister to the wants of the sick of that widely extended district, and also enforced upon them the necessity of sanitary precautions.

When the disease was on the decline in San Lorenzo, I

received an intimation from the priest of the Ajuda district, that his parishioners were falling in great numbers under the disease. I therefore proceeded to that district, taking with me the second class naval surgeon, Jose Maria de Mello Dias, who had just arrived in the island, having previously also given into Mr. Mayer's charge the cases remaining in the San Lorenzo and Nosta Senhora de Conceiçao districts.

The account given by the priest of Monteiros was not exaggerated: the cholera morbus raged there. The whole district and its inhabitants were so terror stricken, that those who fell sick immediately begged for the administration to them of the last sacrament, and looked upon death as certain: some, without any ailment at all, exceptiug their own fears, thought themselves so dangerously ill that they made their wills and gave expression to their last requests,—indeed, so completely was this imaginary with some, that when they were told there was nothing the matter with them, they arose from their beds admitting they were well.

Such was the absence of comforts and resources for the indigent class of this district, and, indeed, of the whole island, that the poor creatures had nothing better for sleeping places than small, cold, damp, one-roomed huts, roofed with straw, the walls of which were made of loose stones and generally without plaster, those same sleeping rooms serving also for the kitchen and the place of deposit of all manner of filth. The bed itself would be a dirty mat, the pillow a bundle of dried grass covered with a cloth, the bed-clothes a sheet, and the diet maize broth. Soon after the arrival of myself and my colleague, this condition of things, which must have increased the deadly character of the disease, was in a measure altered—the sick were supplied with poultry, and the indigent with beef at one penny (fifteen reis) per pound, and the starving were fed gratuitously. Those who had not been attacked by the disease gained courage, and those who were suffering from it began to believe that they were not, as a matter of course, to be victims to it. The number of fatal cases so diminished, that out of one hundred and thirty sick at our arrival only seventeen died, whereas prior to this the mortality had been at the rate of fifty per cent. As soon as the number of new cases had diminished considerably, I entrusted the care of this district and that of S. Katharina, where the epidemy had ceased since the 27th of August, to surgeon Dias.

After journeying through the whole of the island, the better to enable me to arrive at a thorough knowledge of its sanitary condition, I took up my residence in the most central of the districts, where there were still cases occasionally appearing, that I might with the greater facility receive frequent intelligence from my colleagues as to the progress of the disease in their respective districts, and with a view too of being able to give my immediate assistance in case the epidemy broke out in any new quarter.

Upon my arrival in the island, the disease being then in its greatest intensity in the districts of S. Lorenzo, S. Katharina, and Monteiros, I endeavoured to establish hospitals in each of these, that I might have the patients under regular treatment; but I could not, notwithstanding every endeavour, find a house in either of them capable of accommodating even eight or ten sick. If I could have carried out this plan, I am persuaded that the mortality would have been much less, the sick would then have been attended to with greater promptitude and more regularity, their diet would have been different, and when in a state of convalescence they would have been prevented making the dietary mistakes that cost many of them their lives,—such mistakes as eating green beans, maize, and other like substances,—indeed, it was the general belief that these things were not merely innoxious, but that they promoted restoration to health; so that, in reality, the medical man had not only to combat the cholera, but inveterate prejudices too.

Whenever the service required it I visited the different parts of the island, sometimes to assist my colleagues when the disease had broken out or re-appeared, and at other times to inspect the places of interment. In this latter service, my colleague Dias manifested great zeal and activity, not only in covering in the old cemeteries, but in directing and completing the works connected with the new.

The cholera morbus, then, after carrying terror and death into every district of the island, except perhaps the filthiest and generally the least healthy, and certainly the most miserably populated of the whole island—namely, that of the Ribeira do Ilbeo—disappeared. There were no cases in San Katharina after August 27th; in Nostra Senhora da Conceiçao, after September 1st; in San Lorenzo, after September 18th; in Nostra Senhora da Ajuda, after October 4th. Altogether there were 3,508 cases, 643 of which were fatal.

The march of the epidemy, which appeared to me to be always independent of extraneous circumstances, such as more or less heat, the wind, damp, rain, and other atmospheric vicissitudes, was not similar in each district. In San Katharina and Nostra Senhora da Conceiçao, it went through its regular three stages, and terminated for once and all. In Nostra Senhora da Ajuda, it rekindled at the moment when it was thought to be nearly at an end. In San Lorenzo, it reappeared twice; the first and second times, the attacks were graver and more fatal than in the third, when the majority of cases were mild cholerina.

The districts on the sea-shore, and those where the habitations were closer together, forming small villages, were the most severely attacked, and the mortality proportionally greater; and the town of San Philip, the first place attacked, suffered most of all.

The slaves suffered more than any other class, especially those recently imported from Africa; in these the epidemy generally showed itself in the severest form, some of the cases being suddenly fatal. I attribute this in a great measure to absence of all protection from the changes in atmosphere, to a scanty and miserable diet, to the want of anything like care during their sickness, and to the excessive use of ardent spirits, this being obtainable there with facility and in abundance. Indeed, if I may credit what was told me, there was one influential wiseacre who insisted upon it that the country rum *(aqua ardente)* was the best preventive; but, be this as it may, it is a fact that, when I arrived, the heads of families were distributing it abundantly, and even compelling little children to drink it, "et in magnâ quantitate".

The blue stage *(periodo cyanico)* was generally the fatal one; when it was passed, the patient was considered to be safe, unless the reaction took the typhoid *(typhoide)*, the utter prostration of muscular power *(adynamico)*, or the putrid *(ataxico)* character.

I did not meet with any cases where eruptions or glandular swellings of the neck *(parotidas)* supervened. There were many cases of relapse, and they were nearly all fatal. Not taking into account the extremely sudden cases *(casos fulminantes)* which occurred at the first invasion of the disease, and that in some instances had a fatal termination in eight or ten hours, the different stages of the disease then following one upon the other with a marvellous rapidity—not taking these into account, the disease usually ran its course in from three to five days.

I very much desired to make *post mortem* examinations, for I was fully alive to the importance of it; but, unfortunately, neither myself nor my colleagues had the opportunity.

It is pretty generally understood that, in the desire to find a specific, nearly every agent that therapeutics place at our disposal has been tried in combating this disease; but experience shows that no uniform mode of treatment can be successfully followed; and I therefore treated the sick upon the principle of attacking merely the symptoms, using such remedies as the various authorities have most recommended. I observed that, in cases where I used ipecacuanha with saline draughts, in the second stage, with a view to lessen the evacuations, the results were not such as to lead me to prefer this course of treatment to the use of preparations of opium.

The treatment pursued by the islanders themselves, previously to my arrival, was so extraordinary that it deserves mention. In the sudden cases, they administered inward doses of urine mixed with the country rum; and, in the very severe cases, they added to this mixture portions of excrement. Sometimes they used clysters from the decoction of tobacco-leaves, and this without weight or measure! They say that the poor creatures would drink these mixtures, and ask for more; and yet some of them, but not those who were favoured with the tobacco clysters, escaped.

On the 8th of October, the epidemy entirely ceased; and allowing thirty days from thence to elapse, I considered the island free from contagious disease, and my commission consequently ended. I returned to this island, where I arrived on the 17th of November last; and, in bringing to a close this long and but imperfectly drawn report, I have to recommend to your Excellency's notice Guilhame M. Mayer, naval surgeon of the first class; and Jose M. de Mello Dias, naval surgeon of the second class, for the zeal, energy, charitableness, and activity, they displayed during this epidemy. I feel, too, that I should commit a grave fault if I omitted to mention, as worthy of recompense, Mr. Justino Antonio da Silva, high constable for the district of Nostra Senhora da Conceiçao *(ragador da parochia)*, for the important services he rendered.

(Signed) JOSE FERNANDEZ DA SILVA LEAÕ,
*Chirurgeon Mor da Provinci.*

Villa da Praia, January 15, 1856.

